# Supplementary material for: Activation of the epithelial sodium channel (ENaC) leads to cytokine profile shift to pro‐inflammatory in labor
Source: EMBO Mol Med. 2018 Aug 28;10(10):e8868. doi: 10.15252/emmm.201808868 (PMC6402451; doi:10.15252/emmm.201808868)
Supplement: Supplementary file 1 — Appendix [file EMMM-10-e8868-s001.pdf]

Activation of the 1 epithelial sodium channel (ENaC) leads to cytokine profile shift to pro-inflammatory in labor

Xiao Sun, Jing Hui GuoDan Zhang, Jun-jiang Chen, Wei Yin Lin, Yun Huang, Hui Chen, Wen Qing Huang, Yifeng Liu, Lai Ling Tsang, Mei Kuen Yu, Yiu Wa Chung, Xiaohua Jiang, Hefeng Huang, Hsiao Chang Chan and Ye Chun Ruan

Appendix

Appendix Table S1

Appendix Table S1

Figure	Panel	Comparison	p-value
Figure 1	A	16 d.p.c. vs 15 d.p.c.	0.9943
		17 d.p.c. vs 15 d.p.c.	8.991E-07
		18 d.p.c. vs 15 d.p.c.	4E-10
		19 d.p.c. vs 15 d.p.c.	4.7E-14
		1 d.p.p. vs 15 d.p.c.	0.0279
		2 d.p.p. vs 15 d.p.c.	0.0239
	B	15 d.p.c. vs 19 d.p.c.	0.0082
Figure 2	B	Ctrl vs Stretch	6.00E-08
		Stretch vs Stretch+Ami	7.3E-11
	C	(IL-6) DMSO vs DMSO+Stretch	1.89274E-05
		(IL-6) DMSO +Stretch vs Ami+Stretch	0.0011
		(TNFα) DMSO vs DMSO+Stretch	2.86772E-07
		(TNFα) DMSO +Stretch vs Ami+Stretch	3.60872E-05
	D	shNC vs shENaCα	0.0021
	E	(IL-6) shNC vs shNC+Stretch	0.0045
		(IL-6) shNC+Stretch vs shENaC+Stretch	0.0057
		(IL-8) shNC vs shNC+Stretch	0.0002
		(IL-8) shNC+Stretch vs shENaC+Stretch	0.0005
		(TNFα) shNC vs shNC+Stretch	0.0359
		(TNFα) shNC+Stretch vs shENaC+Stretch	0.0003
		(COX-2) shNC vs shNC+Stretch	0.0004
		(COX-2) shNC+Stretch vs shENaC+Stretch	0.0012
	F	Ctrl vs Stretch	0.0005
		Stretch vs Stretch+Ami 10 µM	0.0194
		Stretch vs Stretch+Ami 50 µM	0.0323
	G	Ctrl vs Stretch	0.0006
		Stretch vs Stretch+Ami 10 µM	0.0007
		Stretch vs Stretch+Ami 50 µM	0.0003
	H	(ENaCα/β-tubulin) Ctrl vs Stretch	0.0273
		(Cleaved ENaCα/β-tubulin) Ctrl vs Stretch	0.0196
		(COX-2/β-tubulin)	0.0453

Figure 3	D	Intact vs De-epi	0.0162
	E	DMSO vs Ami 10 μM	0.0176
		DMSO vs Ami 50 μM	0.0047
		DMSO vs Ami 200 μM	0.0001
	F	15 d.p.c. vs 19 d.p.c.	1.45E-06
Figure 4			
	A	ENaCα/β-tubulin (Ctrl vs RU486)	0.0382
		Cleaved ENaCα/β-tubulin (Ctrl vs RU486)	0.0013
	B	Ctrl vs Ami 10 mg/kg	0.0095
		Ctrl vs Ami 20 mg/kg	0.0204
		Ctrl vs Ami 30 mg/kg	0.000032
		Ctrl vs Ami 40 mg/kg	3.3E-09
		Ctrl vs Atosiban	0.0503
	C	siNC vs siENaC	0.0461
Figure 5	B	(ENaCα/β-tubulin)Term vs Preterm	0.0002
		(ENaCβ/β-tubulin)Term vs Preterm	0.2677
		(ENaCγ/β-tubulin)Term vs Preterm	0.1990
Figure EV1		Ctrl vs Stretch	0.015
		Stretch vs Stretch + Ami 10 μM	0.0157
Figure EV3	A	(ENaCα) Ctrl vs LPS	0.18
		(ENaCγ) Ctrl vs LPS	0.0144
	B	LPS vs LPS+ Ami	0.1632
Figure EV4		All_NonPROM vs All_PROM	0.0508
