# Supplementary material for: Activation of the epithelial sodium channel (ENaC) leads to cytokine profile shift to pro‐inflammatory in labor
Source: EMBO Mol Med. 2018 Aug 28;10(10):e8868. doi: 10.15252/emmm.201808868 (PMC6402451; doi:10.15252/emmm.201808868)
Supplement: Supplementary file 2 — Expanded View Figures PDF [file EMMM-10-e8868-s002.pdf]

Expanded View Figures

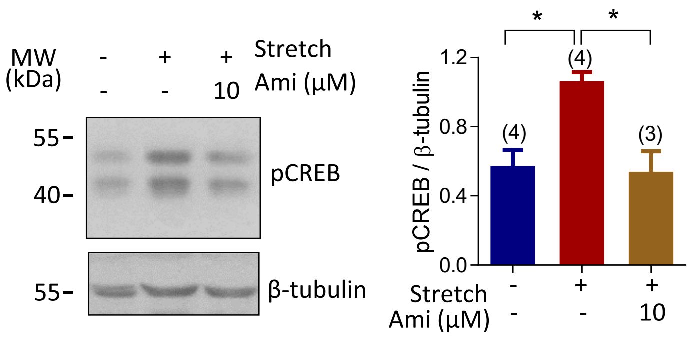

Figure EV1. Stretch induces ENaC-dependent CREB activation in human endometrial epithelial cells.

Western blotting with quantification for pCREB in ISK cells with (+) or without (−) stretch (30 min, 15% elongation), in the absence (−) or presence (+) of Ami (10 μM). Data are shown as mean ± SEM. *n* is indicated in each column. *$P < 0.05$, one-way ANOVA with Tukey's multiple comparisons test. Exact *P*-values are listed in Appendix Table S1.

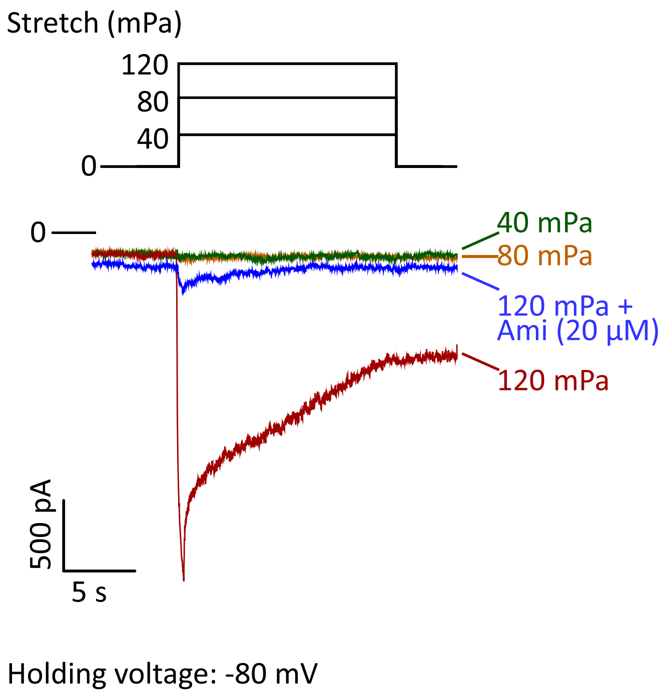

Figure EV2. Mechano-activation of ENaC in human endometrial epithelial cells.

Whole-cell patch-clamp recording of human endometrial epithelial cells at holding voltage of −80 mV when stretches (40–120 mPa) were applied before and after addition of amiloride (Ami, 20 μM).

© 2018 The Authors

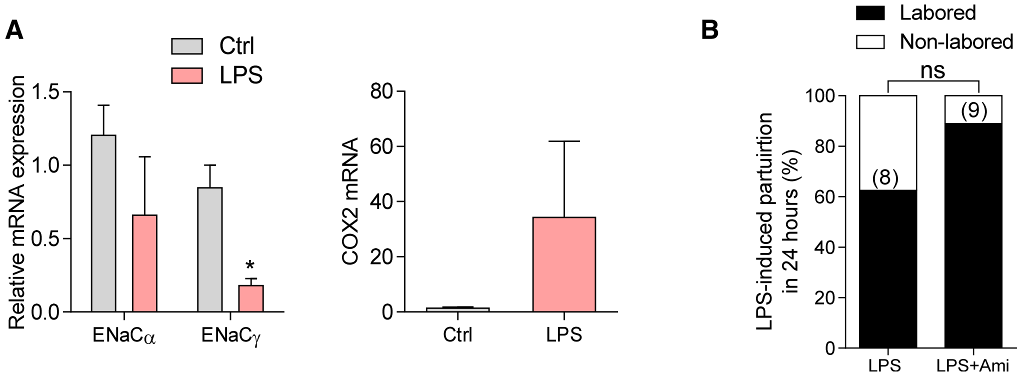

Figure EV3. Effect of amiloride on LPS-induced preterm labor in mice.

A qPCR analysis of ENaCα, γ and COX-2 in LPS (100 μg per mouse, i.p.)-treated mice. Data are shown as mean ± SEM. $n = 3$, $*P < 0.05$, two-tailed unpaired Student's *t*-test.

B Effect of amiloride (Ami, 10 mg/kg body weight, i.p. every 8 h) on LPS-induced preterm labor in mice. Amiloride was injected right before and every 7–8 h after LPS injection cumulatively till the birth of the first pup. Data are percentages of mice labored within 24 h after LPS injection. *n* is shown in each column. ns, not significant, chi-square test.

Data information: Exact *P*-values are listed in Appendix Table S1.

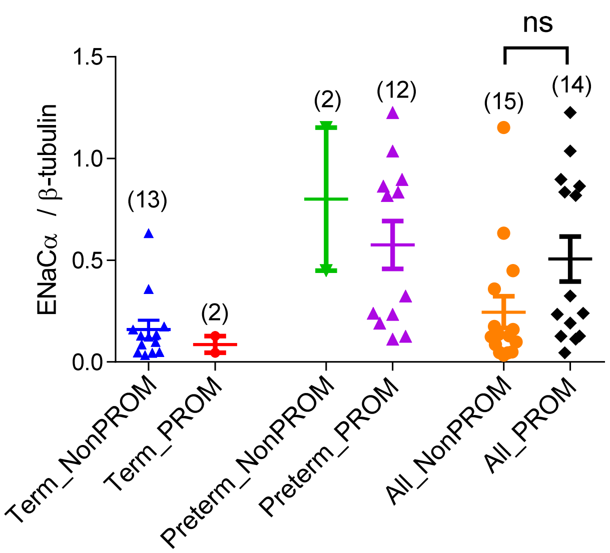

Figure EV4. ENaC expression in women with premature rupture of membrane (PROM).

Quantification of Western blotting for ENaCα (Fig 5) in placental tissues from women labored at term or preterm with or without PROM. Data are shown as mean ± SEM. *n* is indicated for each group. ns: $P > 0.05$, Mann–Whitney test. Exact *P*-values are listed in Appendix Table S1.

© 2018 The Authors

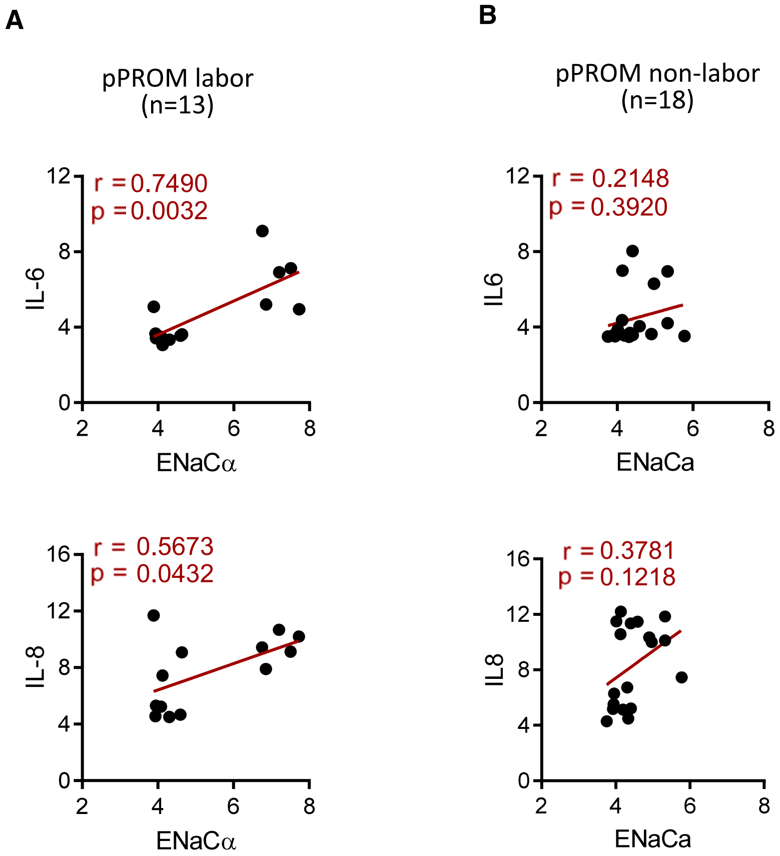

Figure EV5. ENaC is correlated with pro-inflammatory cytokines in women at labor.

A, B Gene expression correlation analysis between ENaCα and IL-6 and IL-8 in human maternal–fetus blood/tissue with preterm premature rupture of membrane (pPROM) labored (A, $n = 13$) or non-labored (B, $n = 18$). The microarray data were retrieved from a human database (Data ref. Baldwin, 2015). n is indicated for each group. Pearson correlation test and values of r and P are shown for each analysis.

 © 2018 The Authors
