# Supplementary figures and images for: Activation of the epithelial sodium channel (ENaC) leads to cytokine profile shift to pro‐inflammatory in labor
Source: EMBO Mol Med. 2018 Aug 28;10(10):e8868. doi: 10.15252/emmm.201808868 (PMC6402451; doi:10.15252/emmm.201808868)

Figure 1B

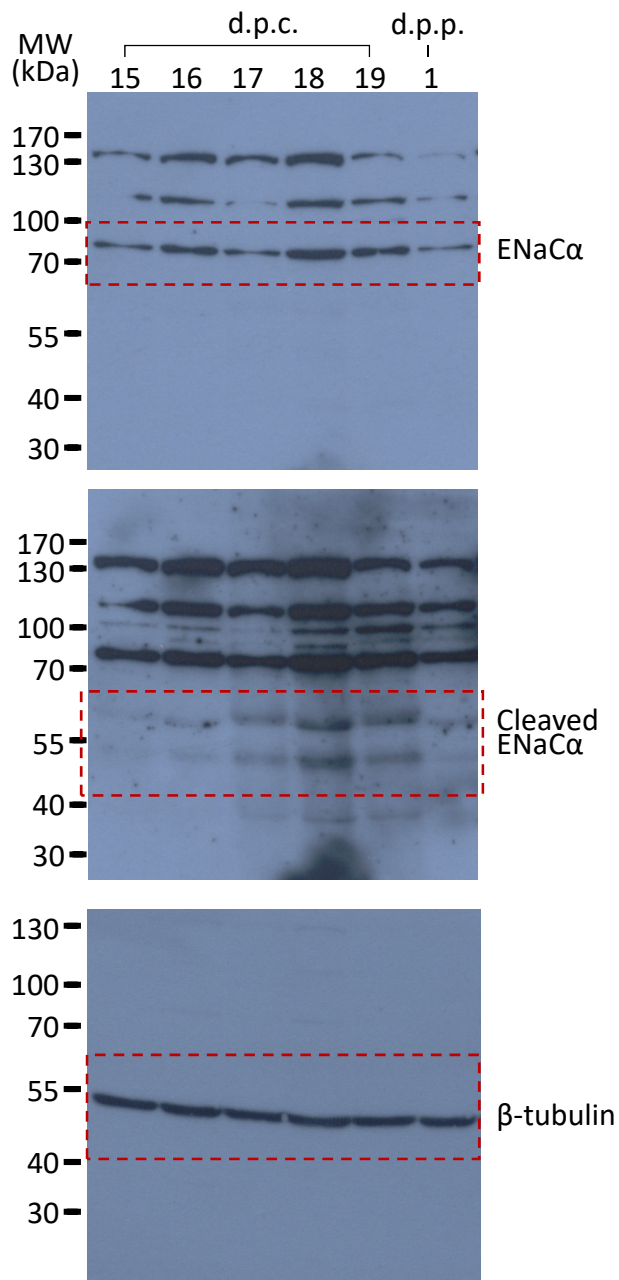

Supplement: Supplementary file 4 — Source Data for Figure 1 [file EMMM-10-e8868-s003.pdf]

Figure 2F

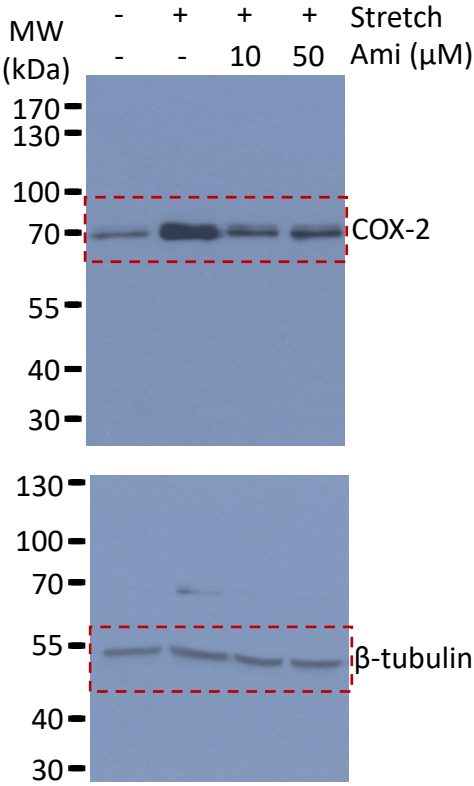

Figure 2H

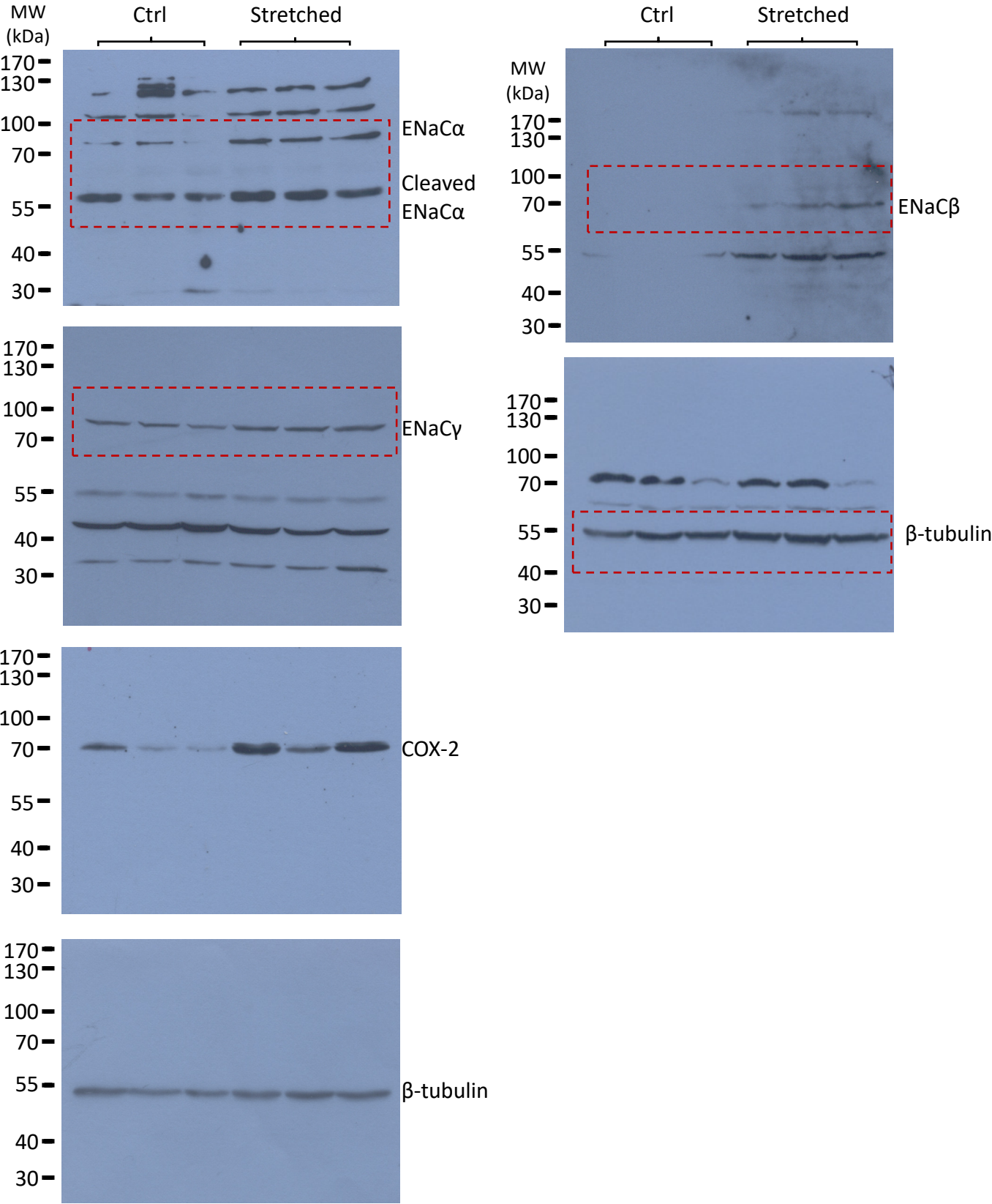

Supplement: Supplementary file 5 — Source Data for Figure 2 [file EMMM-10-e8868-s004.pdf]

Figure 4A

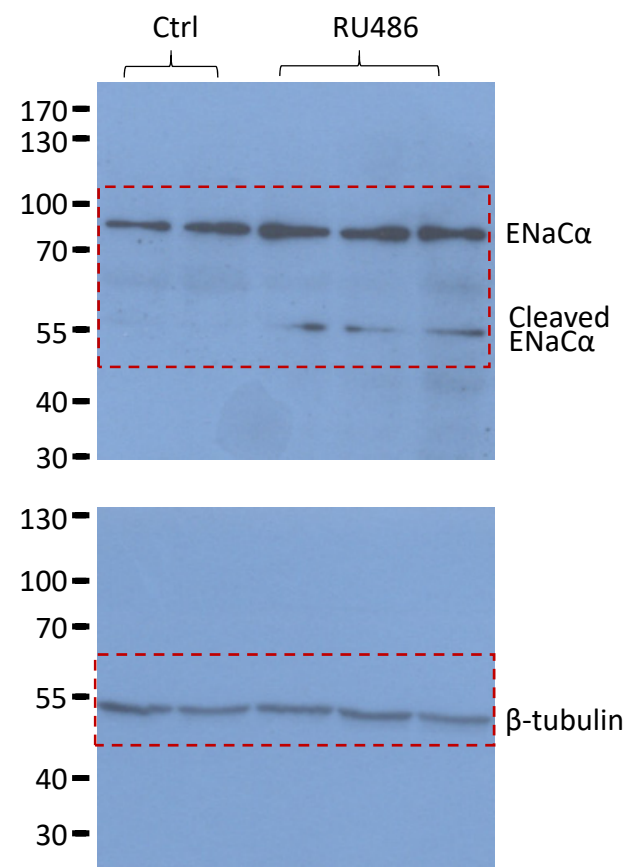

Figure 4C

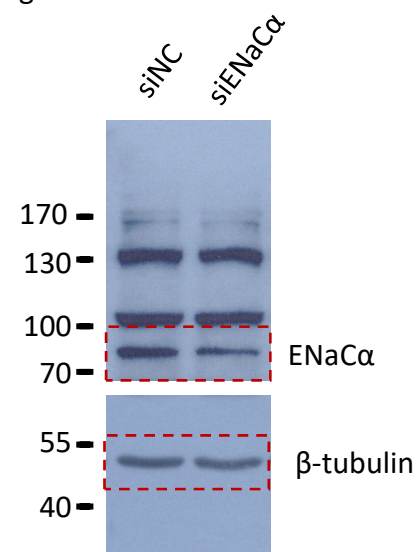

Supplement: Supplementary file 6 — Source Data for Figure 4 [file EMMM-10-e8868-s005.pdf]

Figure 5A

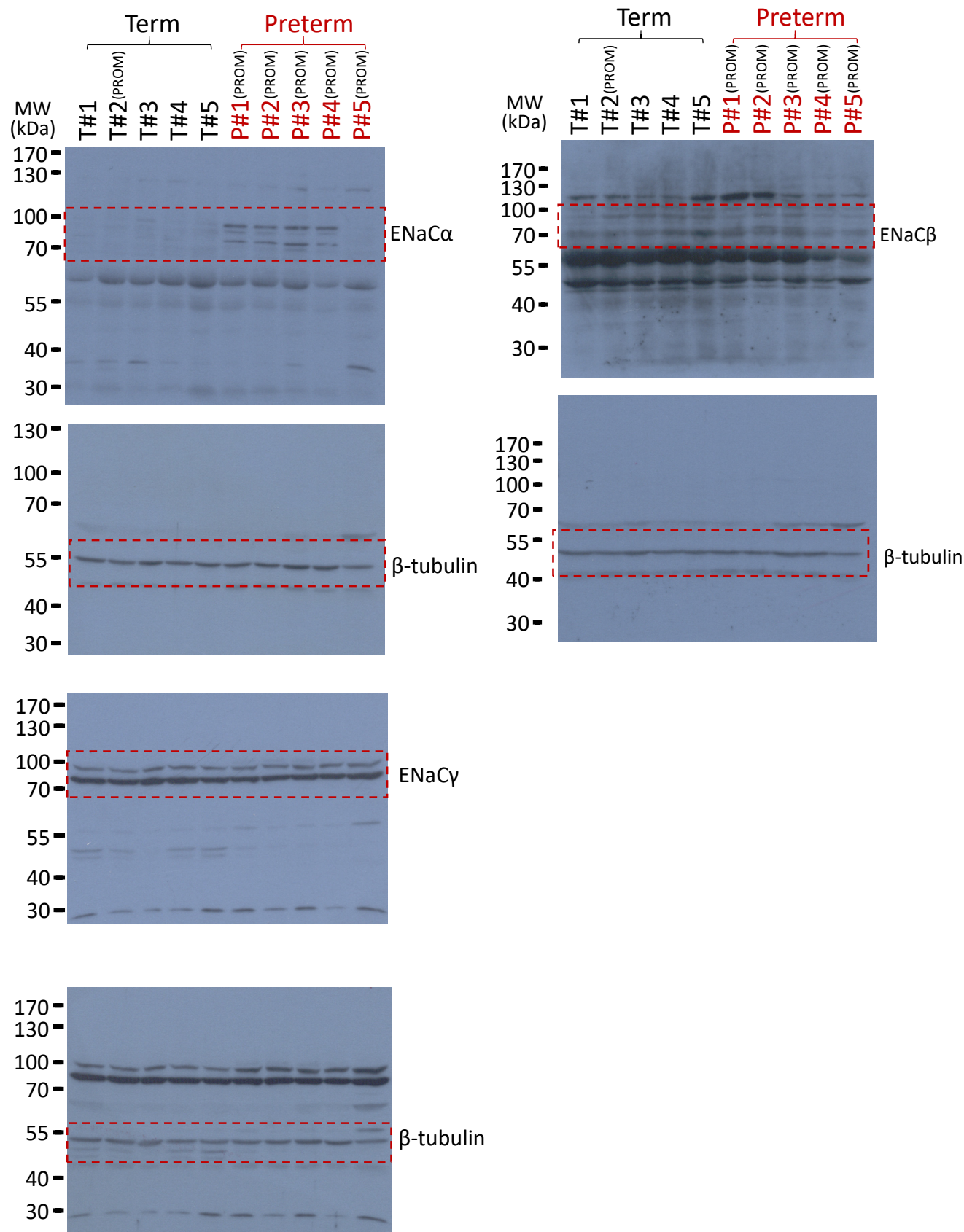

Figure 5A

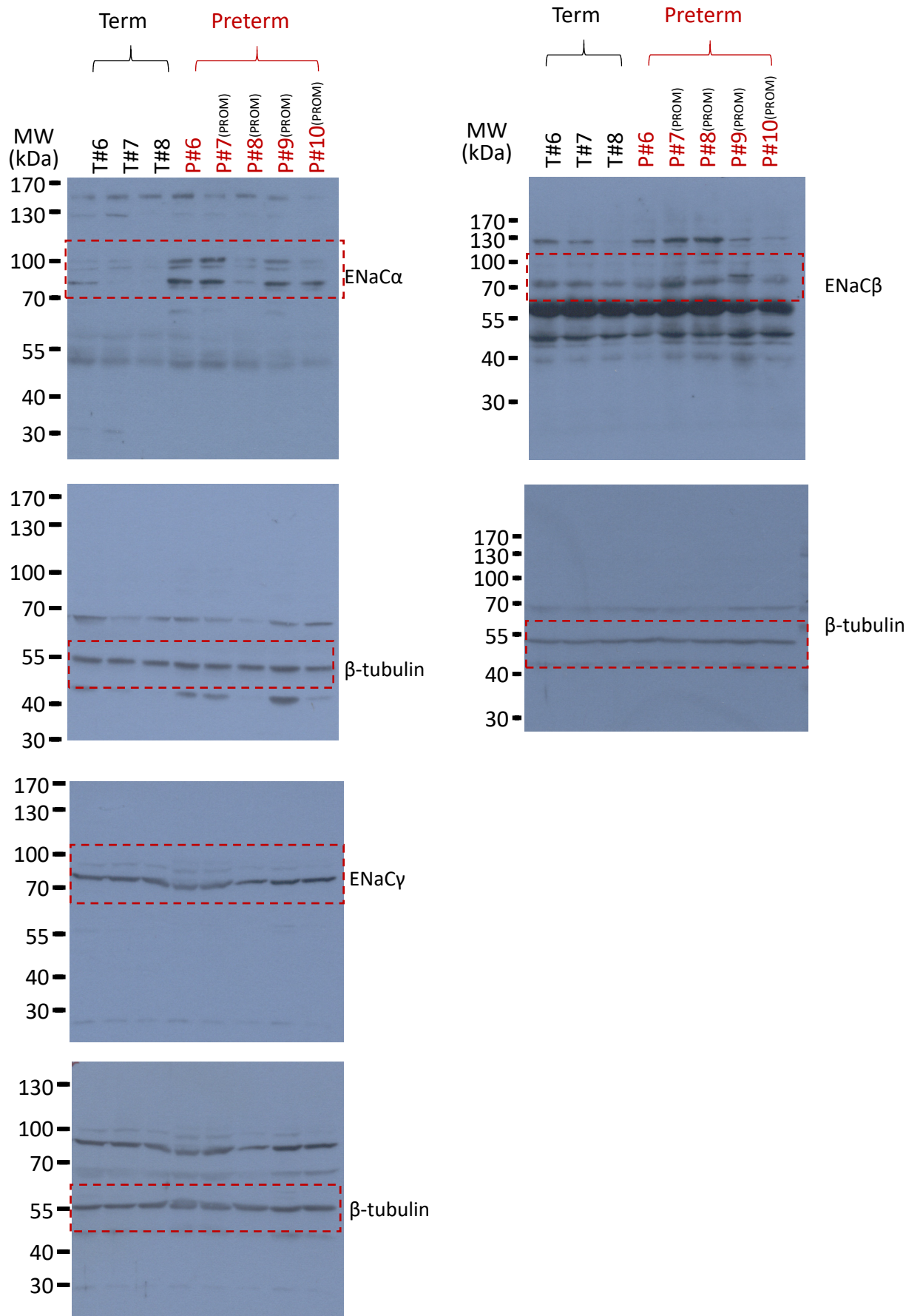

Figure 5A

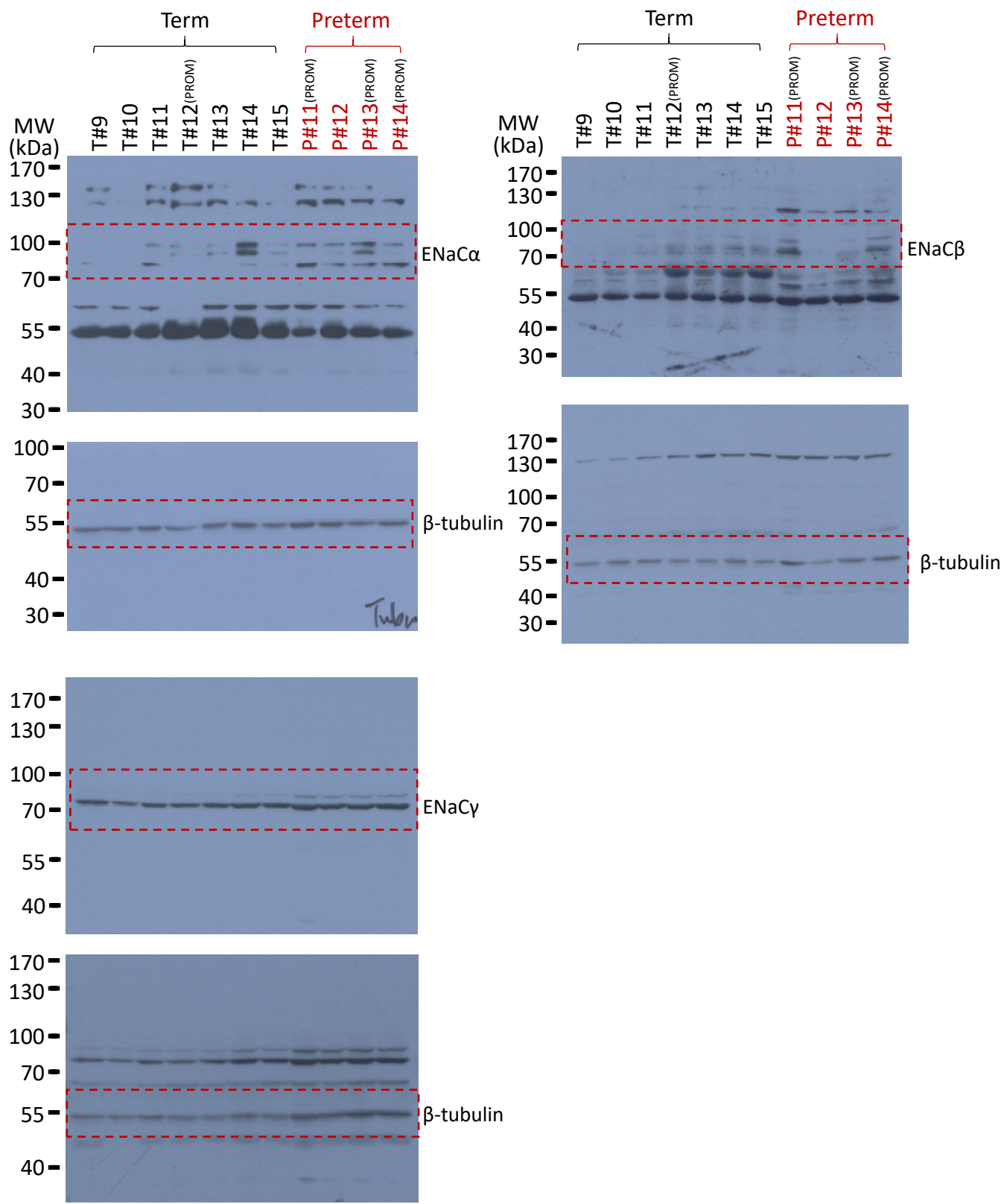

Supplement: Supplementary file 7 — Source Data for Figure 5 [file EMMM-10-e8868-s006.pdf]
